# Multiplex real-time PCR for the detection of *Clavibacter michiganensis* subsp. *michiganensis*, *Pseudomonas syringae* pv. *tomato* and pathogenic *Xanthomonas* species on tomato plants

**DOI:** 10.1371/journal.pone.0227559

**Published:** 2020-01-07

**Authors:** Eliška Peňázová, Miloň Dvořák, Lucia Ragasová, Tomáš Kiss, Jakub Pečenka, Jana Čechová, Aleš Eichmeier

**Affiliations:** 1 Mendeleum–Department of Genetics, Faculty of Horticulture, Mendel University in Brno, Lednice, Czech Republic; 2 Department of Forest Protection and Wildlife Management, Faculty of Forestry and Wood Technology, Mendel University in Brno, Brno, Czech Republic; 3 Department of Vegetable Sciences, Faculty of Horticulture, Mendel University in Brno, Lednice, Czech Republic; University of Helsinki, FINLAND

## Abstract

A multiplex real-time PCR method based on fluorescent TaqMan® probes was developed for the simultaneous detection of the tomato pathogenic bacteria *Clavibacter michiganensis* subsp. *michiganensis*, *Pseudomonas syringae* pv. *tomato* and bacterial spot-causing xanthomonads. The specificity of the multiplex assay was validated on 44 bacterial strains, including 32 target pathogen strains as well as closely related species and nontarget tomato pathogenic bacteria. The designed multiplex real-time PCR showed high sensitivity when positive amplification was observed for one pg of bacterial DNA in the cases of *Clavibacter michiganensis* subsp. *michiganensis* and *Pseudomonas syringae* pv. *tomato* bacteria and 100 pg for bacterial spot-causing xanthomonads. The reliability of the developed multiplex real-time PCR assay for *in planta* detection was verified by recognition of the target pathogens in 18 tomato plants artificially inoculated by each of the target bacteria and tomato samples from production greenhouses.

## Introduction

*Clavibacter michiganensis* subsp. *michiganensis* (Cmm), *Pseudomonas syringae* pv. *tomato* (Pst) and bacterial spot-causing xanthomonads (BSX) represent major bacterial pathogens of tomato [1, 2]. Cmm and BSX are quarantine organisms in the European Union (EPPO A2 list) and are subjected to strict international phytosanitary controls [3, 4]. However, significant losses caused by Pst have also been reported [5, 6].

Cmm (Actinobacteria; family, Microbacteriaceae; genus, *Clavibacter*), the causal agent of bacterial wilt and canker of tomato, is considered one of the most important bacterial pathogens in tomato plantings worldwide. The pathogen infects tomato plants epiphytically through wounds and natural openings such as hydathodes and stomata; however, plants may be infected from infested seeds. Once inside the plant, the pathogen proliferates in xylem vessels, forming extensive biofilm-like structures, which aid in colonization and movement through the plant. Moreover, bacterial ooze from cankers and hydathodes, spread by rain or irrigation water, causes rapid spread to nearbyplants [7–9]. The symptoms of the disease occur on aerial parts and include wilting of leaves and discoloration of vascular tissues resulting in stem canker. At the late stage of infection, black spots with white halos, so-called "bird eyes", may occur on fruit [10, 11].

Bacterial speck of tomato caused by Pst (Proteobacteria; family Pseudomonadaceae; genus *Pseudomonas*) is one of the most persistent bacterial diseases in both the greenhouse and field production of tomatoes. The disease is spread by contaminated tomato seeds and infected weeds in which the bacteria can survive in the weed root system [12, 13]. The pathogen enters the intercellular spaces of leaves through stomata or the base of leaf trichomes and multiplies endophytically and asymptomatically prior to symptom development. The disease is characterized by small necrotic lesions surrounded by chlorotic haloes on the leaves, stems and fruits of tomato plants [5, 6, 14].

Bacterial spot of tomato is caused by multiple species of the genus *Xanthomonas* (Proteobacteria; family Xanthomonadaceae), most recently separated into four distinct species: *X*. *euvesicatoria* (Xe), *X*. *vesicatoria* (Xv), *X*. *perforans* (Xp) and *X*. *gardneri* (Xg) [15]. However, a close evolutionary relationship between Xe and Xp was reported [16, 17]. Among the four species, Xe, Xv and Xg infect both pepper and tomato, while Xp has only been reported on tomato [15]. In 2010, the strain Xp was also isolated from diseased pepper plants by Schwartz *et al*. [18]. Symptoms on tomato leaves are similar to those described for Pst. Small circular lesions sometimes with a yellow halo develop on the leaflets or on the edge of leaves. On immature fruits, dark green to black lesions are formed [19]. The disease is spread through infected seeds, irrigation water, wind or by infected plant debris [20, 21]. Bacteria enter the host plants through stomata on the leaf surfaces or through wounds and colonizethe vascular system [22].

Because of the economic importance of these pathogens, the accurate detection of causal organisms is crucial, and it also represents one of the most effective strategies to prevent their further spread [3, 23]. For pathogen detection, PCR-based methods are the most commonly used. Many conventional PCR protocols for Cmm, Pst and BSX detection were described previously [3, 24–28], as well as real-time PCR assays [29–32] that increased the specificity and sensitivity of PCR detection. Additionally, the use of multiplex reactions has increased the efficiency of laboratories and has led to the faster and more effective identification of the causal organism. In the case of tomato pathogens, conventional multiplex PCR detecting Cmm, Pst and *X*. *axonopodis* pv. *vesicatoria* was described by Özdemir [1]. However, this reaction was optimized on pure bacterial cultures only when bacterial strains ICMP 2550 (Cmm), ICMP 2844 (Pst) and ICMP 9592 (Xe) were tested. The verification of designed multiplex assay on broader spectrum of isolates and diseased plants as well as a possible cross-reaction with nontarget bacteria was not shown. The use of previously described system for Cmm detection through *pat-1* gene carried by plasmid pMC1 [33 bears also the risk of false negative results for isolates lacking this plasmid. Moreover, the separation of PCR products by gel electrophoresis requires more time and higher workload. According to our knowledge, the multiplex real-time PCR for detection of bacteria Cmm, Pst and BSX complex was not described up to now.

The goal of this study was to design a multiplex real-time PCR assay based on TaqMan® probes to achieve a rapid, sensitive and highly specific protocol for *in planta* detection of Cmm, Pst and BSX.

## Materials and methods

### Bacterial strains and plant samples

Bacterial cultures (listed in S1 and S2 Tables) were obtained from the National Collection of Plant Pathogenic Bacteria (NCPPB, London, UK), National Collection of Agricultural and Industrial Microorganisms (NCAIM, Budapest, Hungary), Collection of University of Warwick (HRIW, Wellesbourne, UK) and Crop Research Institute (CRI, Prague, Czech Republic). In total, 44 pathogenic bacterial strains with different geographical origins were tested. The set of samples included 32 strains of target bacteria (six strains of Cmm, eight strains of Pst, 18 strains of BSX), 10 closely related species and two nontarget bacterial pathogens of tomato. The isolates were grown on Mueller-Hinton Agar (HiMedia, Mumbai, India) at 25°C (genus *Pseudomonas*) and 28°C (genera *Clavibacter* and *Xanthomonas*) for 2–3 days prior to DNA extraction.

Positive controls for *in planta* detection were obtained from artificially inoculated tomato seedlings (*Solanum lycopersicum*, cv. Mandat) grown in isolated greenhouse conditions. Reference strains of Cmm (NCPPB 2979), Pst (NCPPB 1106), Xe (NCPPB 2968), Xv (NCPPB 422), Xg (NCPPB 881) and Xp (NCPPB 4321) grown overnight in Mueller-Hinton Broth (HiMedia, Mumbai, India), and bacterial suspensions of approximately 10^8^ CFU.ml^-1^ were prepared in 0.9% sterile physiological saline solution. For each pathogen, 10 one-month-old seedlings were artificially inoculated. The bacterial suspension of Cmm (0.01 ml) was injected into the tomato stems using a sterile needle and syringe [34], and the suspension of Pst and each of the BSX pathogens were sprayed on the leaf surface using a hand atomizer (BOSCH PFS 55, Bosch, Germany) [35]. After the development of symptoms, three plants inoculated with each bacterium were randomly selected; symptomatic parts were used for DNA extraction and pathogen detection. As a negative control, the genomic DNA from healthy plants of seven tomato cultivars (Pedro, Palava, Sonet, Mandat, Curranto, Charmant and Gallant) was used. Seeds and seedlings of all tomato cultivars were provided by the company Moravoseed CZ a. s.

### DNA extraction

Total genomic DNA of bacterial cultures and DNA of tested plants was extracted with the NucleoSpin Tissue kit (Macherey-Nagel, Düren, Germany) according to the manufacturer´s instructions. The DNA of plant samples was isolated from approximately 100 mg of homogenized plant tissue. The DNA concentration of the samples was estimated with a Modulus^™^ Single Tube Multimode Reader (Turner BioSystems, CA, USA) and adjusted to a final concentration of 4–5 ng.μl^-1^ for bacterial cultures and 50 ng.μl^-1^ for the plant samples. A concentration of 50 ng.μl^-1^ was used for nontarget bacterial cultures to increase the probability of their detection in case of nonspecific amplification and to prevent possible false positive results.

### Primer and probe design

Primers and probes (Table 1) were designed using Primer-BLAST [36] and CLC Main Workbench 6.5 (CLC Bio, Aarhus, Denmark) software. The detection system for Cmm used the previously published TaqMan® probe CMM-TP targeting the region of *16-23S rRNA* [29] that allows the differentiation of Cmm from other *Clavibacter michiganensis* subspecies. Newly designed Cmm primers were constructed to be fully compatible with the Cmm probe. For the detection of Pst, the target region of RNA polymerase sigma factor *hrpL* (*hypersensitivity and response pathogenicity*) was used based on its involvement in pathogenicity. The *hrp* gene cluster is considered essential for symptom formation in host plants and for hypersensitive responses in nonhosts. The GTP-binding membrane protein *lepA* (*hypothetical protein for elongation factor 4*) was used as the target for BSX. *LepA* promotes the back translocation of tRNAs on the ribosome during the elongation cycle and has a high conservation in certain bacteria [37, 38, 39], thus allowing highly specific detection. All primers were tested for individual specificity by *in silico* analysis using a Blastn search (GeneBank/NCBI, BLAST 2.2.31+) in which nonredundant collection (nr/nt) and highly similar sequences (megablast) settings were used. The self-complementarity, primer-dimer estimation and melting temperatures of each oligonucleotide were evaluated using Multiple Primer Analyzer (https://www.thermofisher.com/cz/en/home/brands/thermo-scientific/molecular-biology/molecular-biology-learning-center/molecular-biology-resource-library/thermo-scientific-web-tools/multiple-primer-analyzer.html) and OligoAnalyzer Tool (https://www.idtdna.com/pages/tools/oligoanalyzer). For multiplexing, the following combinations of fluorophores and quenchers were used: FAM-BHQ1 (Pst), HEX-BHQ1 (Cmm) and Cy5-BHQ2 (BSX).

**Table 1:** Sequences of primers and probes used in the study.

Primer/probe	Nucleotide sequence (5’-3’ direction)	Target region	Product size
**CMM-16-23S_e_fwd**	GCACCTTCTGGGTGTGTCTG	*16–23 S rRNA*	140 bp
**CMM-16-23S_e_rev**	TGTGATCCACCGGAAAACCG		
CMM TP*	TCCGTCGTCCTGTTGTGGATG (HEX-BHQ1)		
**PST-hrpL_e_fwd**	TTTCAACATGCCAGCAAACC	*hrpL*	169 bp
**PST-hrpL_e_rev**	GATGCCCCTCTACCTGATGA		
**PST-hrpL_TP**	GCTGAACCTGATCCGCAATCAC (FAM -BHQ1)		
**XE_lepA_aec_fwd**	TGATCATCGATTCCTGGT	*lepA*	197 bp
**XE_lepA_cea_rev**	GTTGATCCAGCCCACTTC		
**XE_lepA_TP**	CCAGCGAGACCACGCCCA (Cy5-BHQ2)		

* Designed in [29]

### Optimization of simplex real-time PCR assays

Detection systems were first optimized as simplex assays using DNA from isolates from the NCPPB collection (S1 and S2 Tables) and seven healthy cultivars of tomato. All samples were tested in triplicate on the Roche LightCycler 480 Instrument II (Roche Diagnostics, Basel, Switzerland). The reactions consisted of 10 μl of LightCycler 480 Probes Master (Roche Diagnostics Corp., Basel, Switzerland), 1.2 μl of each of the forward and the reverse primer (10 μM), 0.24 μl of probe (10 μM) and 2 μl of DNA template. The final reaction volume of 20 μl was achieved by the addition of nuclease-free water (Ambion, TFS, Waltham, USA). Annealing temperatures of 60, 62 and 65°C were compared to optimize the thermal profile. The best combination of specificity and reaction efficiency was obtained with an annealing temperature of 65°C. The thermal profile of the reaction was set to 95°C for 10 min followed by 40 cycles of 95°C for 10 s, 65°C for 30 s and 72°C for 1 s. A plate read was set to occur during the 72°C cycle. The thresholds were set automatically according to manually selected noise bands within the assays for each TaqMan® probe.

### Evaluation of assay sensitivity

The sensitivity of the simplex assays was evaluated on serial dilutions of DNA isolated from cultures of Cmm (NCPPB 2979), Pst (NCPPB 1109) and Xe (NCPPB 2689) reference strains. Only Xe was chosen from the BSX complex due to its prevalence in diseased tomatoes. DNA samples were diluted to approximately 50 ng.μl^-1^ with nuclease-free water, and then 10-fold dilution series (5×10^1^–5×10^−4^ ng.μl^-1^) were prepared. All samples were tested in triplicate.

TaqMan® real-time PCR assays were performed on the Roche LightCycler 480 Instrument II (Roche Diagnostics Corp., Basel, Switzerland) using the mastermix LightCycler 480 Probes Master (Roche Diagnostics Corp., Basel, Switzerland) and the thermal profile as described above for the simplex assay optimization. The efficiency of each reaction was determined by a standard curve calculated with qPCR cycler software (LightCycler® 480 Software, Version 1.5.1.62). For the absolute quantification, the fit points method analysis module was used for all the analyses.

### Specificity of the multiplex real-time PCR assay and sensitivity analysis

Once the simplex reaction conditions were optimized, the three assay reactions were multiplexed into one. The triplex assay was performed on the Roche LightCycler 480 Instrument II using 96 multiwell plates. The reaction mixes were comprised of 10 μl of LightCycler 480 Probes Master (Roche Diagnostics Corp., Basel, Switzerland), 1.2 μl of each of the forward and reverse primers (10 μM), 0.24 μl of each probe (10 μM) and 2 μl of DNA template. The final reaction volume of 20 μl was achieved by the addition of nuclease-free water (Ambion, TFS, Waltham, USA). The thermal profile of the reaction was set to 95°C for 10 min followed by 40 cycles of 95°C for 10 s, 65°C for 30 s and 72°C for 1 s. A plate read was set to occur during the 72°C cycle. All samples were tested in triplicate. The multiplex assay was verified on 44 isolates, which are listed in the S1 and S2 Tables. The sensitivity of the assay was evaluated using the same serial dilutions of target bacterial DNAs as in simplex sensitivity analyses.

### Mixed template experiment

Template DNA from Cmm (NCPPB 2979), Pst (NCPPB 1109) and Xe (NCPPB 2689) were mixed together in a 1:1:1 ratio (50 ng.μl^-1^ starting concentrations), and the 10-fold dilution series in the range of 5×10^1^–5×10^−4^ ng.μl^-1^ were tested in simplex and triplex real-time PCR assays. Three replicates of each dilution were used.

### Testing the assay on plant samples from commercial greenhouses

The designed multiplex real-time PCR assay was tested on leaf samples of hydroponic cultured tomatoes originating from greenhouses of two companies—Farm Mutěnice (Mutěnice, Czech Republic, 48.9041283N, 17.0291736E) and Farm Bezdínek (Dolní Lutyně, Czech Republic, 49.8987581N, 18.4281542E). Four tomato cultivars were tested for the presence of Cmm, Pst and BSX: cvs. Axxy (cherry tomato, Farm Bezdínek), Axiany (vine mini-cherry tomato, Farm Bezdínek), Strabena (vine cherry tomato, Farm Mutěnice) and Sweetlette (vine tomato, Farm Bezdínek). Based on the absence of visual symptoms of bacterial infection on tomato plants, samples were chosen randomly–five plants per cultivar. The presence of target pathogens was tested by a designed multiplex real-time PCR assay and evaluated by cultivating samples on nutrient media specific for Cmm, Phyto Cmm Agar Base (HiMedia, Mummbai, India); Pst, Phyto Pst Agar Base (HiMedia, Mummbai, India); and BSX, Phyto Xcv Agar Base (HiMedia, Mumbai, India) enriched by supplements according to the manufacturer´s instructions. Briefly, leaves suspected for pathogen presence were surface sterilized with 0.5% sodium hypochlorite and four pieces with approx. sizes of 1 cm^2^ were placed into 1 ml of physiological solution (0.9% NaCl) for 15 min. For plating on media, 50 μl of the extract was used.

## Results

### *In silico* analysis of primers and probe specificity

Oligonucleotide primers were first tested for formation of self-dimers and primer-dimers. According to the Multiple Primer Analyzer tool, self-dimers do not form. The possible primer-dimers are presented in S1 Fig. The specificity of designed primers was tested by *in silico* analysis using a Blastn search (GenBank/NCBI). Designed sequences were compared with sequences available in the GenBank database using a threshold of 95% similarity. The organisms possibly amplified by the designed oligonucleotides are presented in S3 Table. The *in silico* analyses showed that the combination of oligonucleotides for Cmm and Pst detected only the target organisms. The primers and probe used for BSX detection showed the possible amplification of five other bacteria, *Xanthomonas axonopodis* pv. *commiphorae*, *Xanthomonas campestris* pv. *arecae*, *Xanthomonas campestris* pv. *musacearum*, *Xanthomonas fuscans* subsp. *fuscans* and *Xanthomonas vasicola* pv. *vasculorum*. However, these pathogens have not been described on tomato plants.

Designed sequences were also compared with partial or complete genome sequences of target bacteria available in GenBank. The primers and probe designed for Cmm detection had 100% identity with nucleotides from the 16S rRNA and 16S-23S rRNA intergenic spacer of isolates from Chile (PBC A4755, Pacific Bacterial Collection), China (PBC A4758), the Netherlands (PBC A5131), South Korea (TF2644), the United Kingdom (NCPPB 382) or the USA (NCPPB 870). A substitution of one nucleotide in the forward primer sequence (8^th^ position) and a complete match in the reverse primer and probe sequence was observed for isolates originating in China (PBC A4757), Kenya (NCPPB 170), Italy (NCPPB 1064) and Morocco (PBC A4763). A similar observation was made for *lepA* gene sequences of xanthomonads from the BSX complex. There was 100% identity for the probe and reverse primer sequences with a one mismatch in the forward primer (14^th^ nucleotide) for strains originating from Argentina (LMG 159), Costa Rica (NCPPB 4323), Hungary (LMG 159), New Zealand (LMG 509) or Zimbabwe (LMG 424). In the case of *Pseudomonas syringae* pv. *tomato*, designed oligonucleotides completely corresponded to the *hrpL* sequences of strains originating from the USA (DC3000, isolate NCPPB 2968) and the United Kingdom (MAFF 302272, Ministry of Agriculture, Fisheries and Food, Japan). The substitution of two nucleotides in the forward and one nucleotide in the reverse primer sequence was found in the case of Canadian Pst strain B13-200.

### Specificity and sensitivity of simplex real-time PCR

Detection systems were tested on DNA from the isolates listed in S1 and S2 Tables and seven healthy cultivars of tomato. At the annealing temperature of 65°C, only strains *X*. *axonopodis* pv. *phaseoli* and *X*. *hortorum* pv. *carotae* showed nontarget amplification in the assay for BSX detection (S4 Table).

The sensitivity of the simplex reaction was evaluated on serial dilutions of genomic DNA purified from Cmm (NCPPB 2979), Pst (NCPPB 1106) and Xe (NCPPB 2689) (Table 2). The Pst detection assay exhibited consistent amplification of the *hrpL* gene from 1 pg of bacterial genomic DNA in the reaction. The detection sensitivity for Cmm and Xe was 10 times lower. The reaction efficiency was calculated as 99.3% for Cmm, 93.4% for Pst and 99.6% for BSX detection. The detection of BSX through the *lepA* gene showed consistently higher Ct values than the assays for Cmm and Pst detection.

**Table 2:** Sensitivity analysis of simplex assays for serial dilution of genomic DNA from pathogenic Cmm, Pst and Xe. Mean Ct values are presented.

Amount of DNA per reaction	HEX (Cmm NCPPB 2797)	FAM (Pst NCPPB 1106)	CY5 (Xe NCPPB 2689)
**100 ng**	15.71	16.75	21.52
**10 ng**	19.02	19.49	24.53
**1 ng**	22.55	23.10	26.94
**0.1 ng**	25.61	26.09	31.82
**0.01 ng**	29.11	30.36	33.50
**0.001 ng**	-	33.31	-
**Efficiency (%)**	99.3	93.4	99.6
**Slope**	-3.340	-3.490	-3.331
**Y-intercept**	22.40	22.99	27.87

### Specificity of multiplex real-time PCR assay

The multiplex assay was tested on the bacterial isolates listed in S1 and S2 Tables. The specificity of the designed primers and probes was tested on 32 target bacteria, 10 closely related species (*C*. *m*. subsp. *insidiosus*, *C*. *m*. subsp. *tesselarius*, *P*. *s*. pv. *syringae*, *X*. *axonopodis* pv. *phaseoli*, *X*. *campestris* pv. *armoraciae*, *X*. *c*. pv. *campestris*, *X*. *c*. pv. *incanae*, *X*. *c*. pv. *raphani*, *X*. *cucurbitae* and *X*. *hortorum* pv. *carotae*) and two nontarget tomato pathogens (*Pseudomonas corrugata*, *Pectobacterium carotovorum* subsp. *carotovorum*). Positive detection of the target strains was achieved for all TaqMan® probes (Table 3). The assay for the identification of Cmm (HEX channel) showed positive results for all virulent and avirulent (NCPPB 515) strains of Cmm. The closely related subspecies *C*. *m*. subsp. *insidiosus* and *C*. *m*. subsp. *tesselarius* were not amplified. However, amplification was observed at ˃37 cycles for CRI 211 (Pst) and NCPPB 281 (*Pectobacterium carotovorum* subsp. *carotovorum*). Therefore, a cycle cut‐off of 37 was established as the highest value for reliable detection.

**Table 3:** The mean Ct values for each fluorescent TaqMan® probe obtained from the designed multiplex real-time PCR assays. Mean Ct values ± standard deviation are presented.

Target bacteria	Isolate	HEX	FAM	Cy5
***C*. *m*. subsp. *michiganensis***	NCPPB 515	20.63 ± 0.08	-	-
	NCPPB 1064	21.43 ± 0.05	-	-
	NCPPB 1496	21.27 ± 0.04	-	-
	NCPPB 2323	21.34 ± 0.06	-	-
	NCPPB 2979*	20.89 ± 0.01	-	-
	NCPPB 3120	21.56 ± 0.03	-	-
***P*. *s*. pv. *tomato***	NCPPB 878	-	-	-
	NCPPB 1106*	-	22.01 ± 0.67	-
	NCPPB 2683	-	24.71 ± 0.15	-
	NCPPB 3333	-	22.43 ± 0.26	-
	NCPPB 3787	-	24.09 ± 0.62	-
	NCPPB 4369	-	21.72 ± 0.39	-
	CRI 111	-	23.33 ± 0.49	-
	CRI 211	37.87 ± 1.12	23.69 ± 0.05	-
***X*. *euvesicatoria***	NCPPB 941	-	-	23.46 ± 0.25
	NCPPB 2574	-	-	23.40 ± 0.66
	NCPPB 2594	-	-	23.03 ± 0.47
	NCPPB 2968*	-	-	23.19 ± 0.13
***X*. *vesicatoria***	NCPPB 422*	-	-	22.51 ± 0.20
	NCPPB 1421	-	-	23.26 ± 0.84
	NCPPB 2044	-	-	22.75 ± 0.04
	NCPPB 3786	-	-	24.82 ± 0.45
***X*. *gardneri***	NCPPB 881*	-	-	21.86 ± 0.51
***X*. *perforans***	NCPPB 4321*	-	-	21.97 ± 0.04
***X*. *a*. pv. *vesicatoria***	CRI 1008	-	-	24.42 ± 0.13
	CRI 1009	-	-	24.52 ± 0.13
	CRI 1011	-	-	24.38 ± 0.08
	CRI 1013	-	-	24.21 ± 0.10
	CRI 1016	-	-	24.23 ± 0.27
	CRI 1018	-	-	24.39 ± 0.28
	CRI 1023	-	-	24.54 ± 0.17
	CRI 1026	-	-	23.77 ± 0.10
**Nontarget bacteria**				
***C*. *m*. subsp. *insidiosus***	NCPPB 1109*	-	-	-
***C*. *m*. subsp. *tesselarius***	NCPPB 3664*	-	-	-
***P*. *s*. pv. *syringae***	NCPPB 2750	-	-	-
***P*. *corrugata***	NCPPB 2445*	-	-	-
***P*. *c*. subsp. *carotovorum***	NCPPB 281*	37.07 ± 0.83	-	-
***X*. *a*. pv. *phaseoli***	NCAIM B.01695	-	-	23.93 ± 0.04
***X*. *c*. pv. *armoraciae***	NCAIM B.01281	-	-	-
***X*. *c*. pv. *campestris***	NCPPB 528*	-	-	-
***X*. *c*. pv. *incanae***	HRIW 6377	-	-	-
***X*. *c*. pv. *raphani***	HRIW 8503	-	-	-
***X*. *cucurbitae***	NCAIM B.01397	-	-	-
***X*. *h*. pv. *carotae***	NCAIM B.01586	-	-	24.89 ± 0.08

* Reference isolate

**Fig 1:**
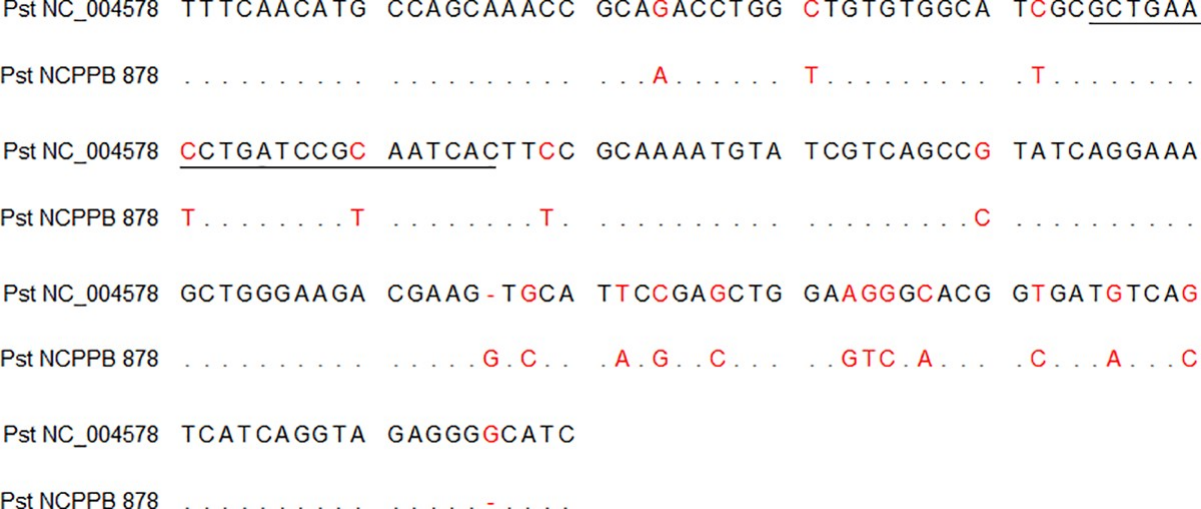
Comparison of partial *hrpL* sequences of the Pst reference isolate (NC_004578) and isolate NCPPB 878. The underlined area shows the hybridization site of the TaqMan® probe for the designed assay, and the red letters show differences in nucleotides.

The assay for Pst identification (FAM channel) successfully amplified seven out of eight Pst isolates, although detectable fluorescence was not recorded for Pst strain NCPPB 878. The sequence amplified from NCPPB 878 with *hrpL* primers in conventional PCR showed variability at the hybridization site of the TaqMan® probe in two positions where a nucleotide substitution of T instead of C was present in the Pst reference sequence (NC_004578) (Fig 1). Nonspecific amplification was not observed for the other bacteria.

Primers and the probe designed for the detection of the *lepA* gene of BSX pathogens (Cy5 channel) proved reliable for identification of all BSX members, *X*. *euvesicatoria*, *X*. *vesicatoria*, *X*. *gardneri* and *X*. *perforans*, as well as for the *X*. *axonopodis* pv. *vesicatoria* strains. Nonspecific amplification was observed only in the case of *X*. *axonopodis* pv. *phaseoli* and *X*. *hortorum* pv. *carotae*. The reactions with the other bacteria showed negative results.

No amplification occurred from all samples of plant genomic DNA extracted from healthy tomato plants.

### Sensitivity of the designed multiplex real-time PCR assay

The sensitivity of the multiplex real-time PCR exhibited consistent amplification of the *16S rRNA* and *hrpL* genes from 1 pg of genomic DNA in the reaction (Table 4). The sensitivity of detection was 100 times lower for the of *lepA* gene compared to the *16S rRNA* and *hrpL* genes. The reaction efficiency reached values of 97.8% for Cmm, 95.5% for Pst and 99.3% for BSX detection.

**Table 4:** Sensitivity analysis of the multiplex assay for serially diluted genomic DNA of pathogenic Cmm, Pst and Xe. Mean Ct values are presented.

Amount of DNA per reaction	HEX (Cmm NCPPB 2797)	FAM (Pst NCPPB 1106)	CY5 (Xe NCPPB 2689)
**100 ng**	17.52	18.23	19.82
**10 ng**	20.89	21.54	23.36
**1 ng**	24.28	25.72	26.50
**0.1 ng**	27.67	29.50	31.13
**0.01 ng**	31.02	31.63	-
**0.001 ng**	34.39	35.39	-
**Efficiency (%)**	97.8	95.5	99.3
**Slope**	-3.375	-3.424	-3.340
**Y-intercept**	24.27	25.30	34.47

### Mixed template experiment and *in planta* detection

**Fig 2:**
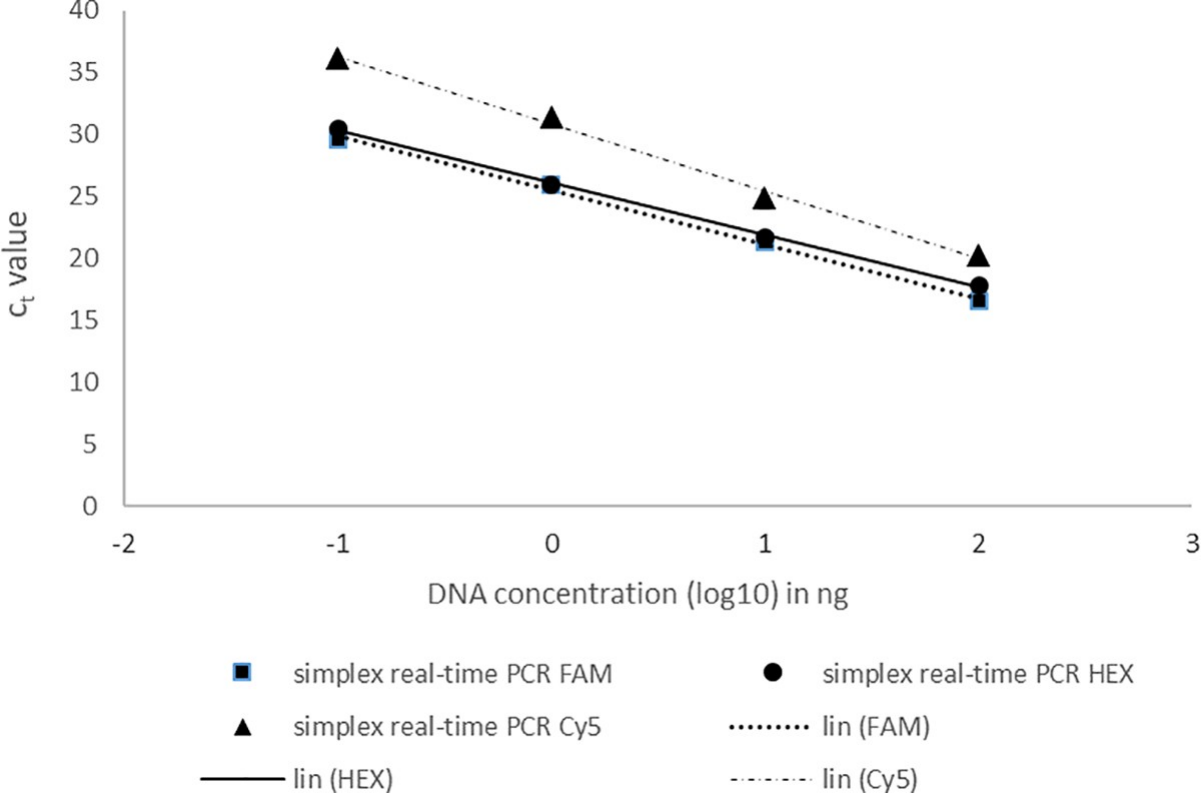
Detection of pathogenic Cmm, Pst and Xe in mixed samples by simplex real-time PCR assays. Log_10_ values of the starting mixed DNA concentrations are plotted against the corresponding Ct values. Each dot represents data from triplicate TaqMan real-time PCR amplifications.

**Fig 3:**
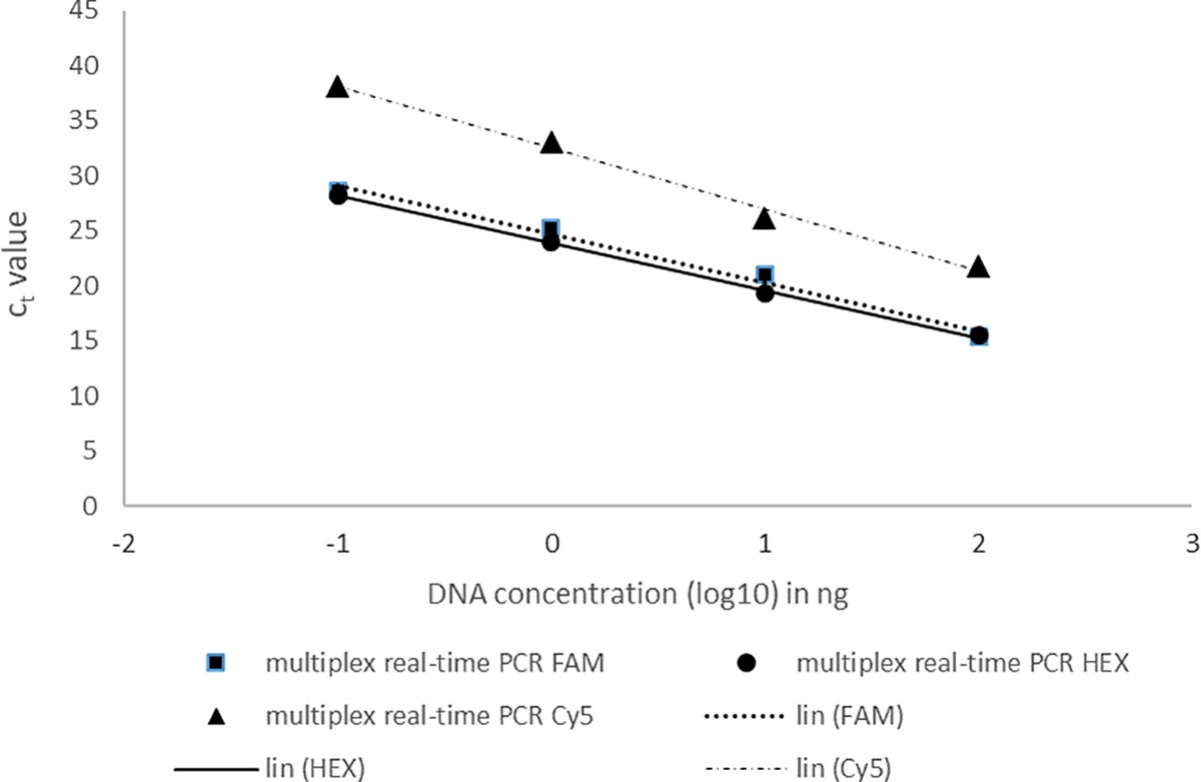
Detection of pathogenic Cmm, Pst and Xe in mixed samples by multiplex real-time PCR assays. Log_10_ values of the starting mixed DNA concentrations are plotted against the corresponding Ct values. Each dot represents data from triplicate amplifications.

The DNA of Cmm, Pst and Xe mixed at a ratio of 1:1:1 was diluted in a 10-fold dilution series and tested by the real-time PCR assay. Positive results were obtained up to the 1000-fold dilution in both simplex and multiplex assays (Figs 2 and 3), which represented the amount of 1 pg of genomic DNA in the reaction. In the case of the 1000-fold dilution, the Cmm (HEX) and Pst (FAM) detection exhibited mean Ct values of approximately 30 in both the simplex and multiplex reactions. The Ct values for Xe detection were higher than those obtained for Cmm and Pst detection. The mean Ct values for Xe detection were 36 in the simplex reaction and 38 in the multiplex reaction.

The *in planta* detection of the designed assay was verified on artificially inoculated one-month-old tomato plants. The target pathogens were successfully detected by multiplex real-time PCR assay using *16-23S rRNA*, *hrpL* and *lepA* genes on appropriate channels in all tested samples (Table 5).

**Table 5:** Detection of pathogenic Cmm, Pst and BSX by multiplex real-time PCR in artificially inoculated plant samples.

Sample	FAM (Pst)	HEX (Cmm)	Cy5 (BSX)	Sample	FAM (Pst)	HEX (Cmm)	Cy5 (BSX)
**Pst1**	29.23 ± 0.33	-	-	**Xv1**	-	-	36.47 ± 0.18
**Pst2**	27.01 ± 0.16	-	-	**Xv2**	-	-	33.88 ± 0.67
**Pst3**	24.59 ± 0.21	-	-	**Xv3**	-	-	36.03 ± 0.17
**Cmm1**	-	24.21 ± 0.03	-	**Xg1**	-	-	32.20 ± 0.28
**Cmm2**	-	26.83 ± 0.20	-	**Xg2**	-	-	33.75 ± 0.42
**Cmm3**	-	21.24 ± 0.42	-	**Xg3**	-	-	31.85 ± 0.29
**Xe1**	-	-	25.53 ± 0.52	**Xp1**	-	-	30.62 ± 0.76
**Xe2**	-	-	24.64 ± 0.16	**Xp2**	-	-	30.86 ± 0.16
**Xe3**	-	-	25.45 ± 0.34	**Xp3**	-	-	31.55 ± 0.47

### Testing the designed multiplex assay on greenhouse samples

Leaf samples of four tomato cultivars randomly collected from plants in production greenhouses were tested for the presence of pathogenic Cmm, Pst and BSX by the designed multiplex real-time PCR assay. None of the 20 samples showed positive detection of the target organisms. The presence of pathogens was also tested by cultivation of leaf extracts on selective media, but characteristic colonies were not observed.

## Discussion

This study provides the first report of a multiplex TaqMan® real-time PCR assay that simultaneously targets the Cmm, Pst and BSX complex. For the detection of these pathogens, conventional multiplex PCR was optimized by Özdemir [1] using previously published systems for Cmm [33], Pst [40 and Xav [41] detection. However, this system was tested only on three cultures, Cmm ICMP 2550 (NCPPB 2979), Pst ICMP 2844 (NCPPB 1106) and Xav ICMP 9592, which were recently classified as *X*. *euvesicatoria* [15]. Additionally, the specificity analyses were confirmed neither on a broader spectrum of target and nontarget isolates nor on plant samples. Because Cmm and BSX are quarantine organisms, the use of real-time PCR methods is more accurate for providing information about the health of tested material.

Real-time PCR assays using TaqMan® probes for Cmm detection were previously published [29, 42, 43]. Available detection systems use the target sequence of the internal transcribed spacer (ITS) located between the 16S and 23S rDNA, as in this study. This region is generally used to avoid possible false-negative results that can be reported when plasmid-borne genes encoding pathogenicity (*pat-1* and *celA*) are targeted [44, 45]. The specificity of the designed assay for Cmm was confirmed by the specific detection of *C*. *michiganensis* and both virulent and avirulent strains (Table 3). Nevertheless, based on the nonspecific amplification of *Pectobacterium carotovorum* subsp. *carotovorum* and Pst isolate CRI 211, the cycle cut-off value of Ct 37 is recommended. The application of a cut-off value in qPCR protocols is frequently used by diagnostic laboratories to prevent false positive results [46]. The qPCR system targeting putative two-component system sensor kinases [47] for Cmm identification that was recommended by EPPO [48] also applies the limit of Ct 35. This protocol was not used in this study because of the possible hybridization of the primers RZ_ptssk 10/RZ_ptssk 11 with Pst isolates, as a difference in two nucleotides between both primers and the Pst reference sequence was found by *in silico* analysis.

The designed system for the identification of Pst pathogens targets the *hrpL* gene from the *hrp* cluster, which was reported as suitable for PCR-specific detection [25, 26]. The *hrpL* gene encodes the alternative sigma factor required for the expression of the *hrp* gene cluster in the *Pseudomonas syringae* group [49, 50], which is essential for symptom development in host plants and the hypersensitive response in nonhosts [51, 52]. The specificity of the reaction was proven for seven of eight tested isolates of Pst. Nonspecific amplification was not observed. In the case of strain NCPPB 878, lack of detection was caused by nucleotide substitutions at the probe hybridization site. However, the nucleotide sequence of the target locus of the *hrpL* gene showed 97% identity with *Pseudomonas syringae* pathovars *aptata*, *panici*, *pisi* and *syringae* but only 88% identity with the Pst reference sequence (GenBank Acc. No. NC_004578) when the Blastn search (BLAST 2.2.31+) was applied. Additionally, the NCPPB collection does not declare the authenticity of isolate 878, although the origin from *S*. *lycopersicum* and its pathogenicity was confirmed by NCPPB.

The published real-time PCR protocols for BSX pathogens are focused mainly on the distinction of the involved species [32, 53]. Since the worldwide distribution of *X*. *perforans* [54] and *X*. *gardneri* [55] was reported, the presence of all BSX species should be tested. In the detection of the BSX group, the *lepA* gene was successfully amplified from all four species and natural isolates from the CRI collection. Nonspecific amplification was obtained only for the isolates *X*. *axonopodis* pv. *phaseoli* and *X*. *hortorum pv*. *carotae*. Based on the *in silico* analyses of the designed oligonucleotides, the amplification of *X*. *a*. pv. *phaseoli* should be prevented by incompatibility with the BSX probe. Nevertheless, none of these pathogens were reported on tomato plants; thus, a false positive result is not expected.

In sum, the designed assay proved high specificity in both simplex and multiplex reactions. The specificity of the multiplex assay was not influenced by the involvement of the primers and probes used except for *Pectobacterium carotovorum* subsp. *carotovorum* and *Xanthomonas axonopodis* pv. *phaseoli*, which were detected by the assay designed for BSX. The reliable results were also obtained when target DNAs were mixed and 1000 times diluted to the final amount of approx. 0.1 ng of genomic/total DNA per reaction. The verification of plant samples confirmed the usability of the designed detection assay, which allows the recognition of quarantine organisms. The negative result for samples from commercial greenhouses confirmed the high level of phytosanitary practice that is required for the ecological approach to be applied by concerned companies. The verification of the designed assay on seeds was not performed based on the quarantine character of pathogenic Cmm and BSX and strict sanitary controls that created the unavailability of infected seeds for this study. Nevertheless, this multiplex real-time PCR based on three TaqMan® probes was shown to be a useful tool for the quick, specific and sensitive detection of the quarantine and economically important tomato bacterial pathogens *Clavibacter michiganensis* subsp. *michiganensis*, *Pseudomonas syringae* pv. *tomato* and bacterial spot-causing xanthomonads in both bacterial cultures and tomato plants.

## Supporting information

S1 Fig Results for primer dimer detection. (TIF)

S1 Table Target bacterial cultures used in the study. (DOC)

S2 Table Nontarget bacterial cultures used in the study. (DOC)

S3 Table In silico analysis of the specificity of designed oligonucleotides using Blastn search (NCBI/GenBank). The significant similarity of nontarget organisms up to 95% is shown.
(DOC)

S4 Table Ct values (mean value ± SD) obtained for the simplex real-time PCRs. (DOC)
