# Supplementary figures and images for: Multiplex real-time PCR for the detection of Clavibacter michiganensis subsp. michiganensis, Pseudomonas syringae pv. tomato and pathogenic Xanthomonas species on tomato plants
Source: PLoS One. 2020 Jan 7;15(1):e0227559. doi: 10.1371/journal.pone.0227559 (PMC6946519; doi:10.1371/journal.pone.0227559)

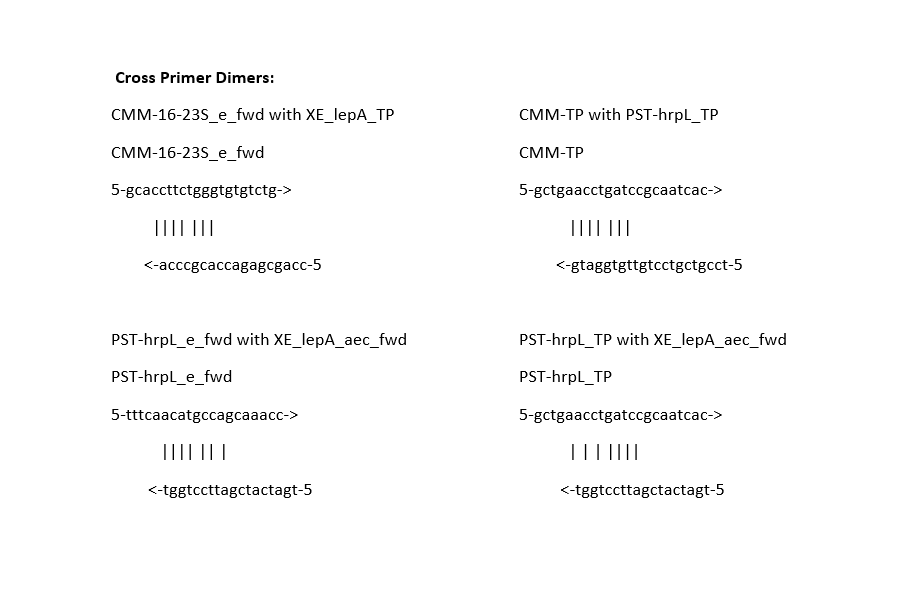

Supplement: S1 Fig — (TIF) [file pone.0227559.s001.tif]
