# Supplementary material for: Multiplex real-time PCR for the detection of Clavibacter michiganensis subsp. michiganensis, Pseudomonas syringae pv. tomato and pathogenic Xanthomonas species on tomato plants
Source: PLoS One. 2020 Jan 7;15(1):e0227559. doi: 10.1371/journal.pone.0227559 (PMC6946519; doi:10.1371/journal.pone.0227559)
Supplement: S1 Table — (DOC) [file pone.0227559.s002.doc]

**Supporting information**

**S1 Table. Target bacterial cultures used in the study.**

| **Bacterium** | **Strain** | **Host** | **Origin** | **Year of isolation** |
| --- | --- | --- | --- | --- |
| *Clavibacter michiganensis* subsp. *michiganensis* | NCPPB 515 | *Solanum lycopersicum* | Italy | 1957 |
|  | NCPPB 1064 | *S. lycopersicum* | Italy | 1961 |
|  | NCPPB 1496 | *-* | - | 1963 |
|  | NCPPB 2323 | *S. lycopersicum* | Romania | 1970 |
|  | NCPPB 2979* | *S. lycopersicum* | Hungary | 1977 |
|  | NCPPB 3120 | *S. lycopersicum* | UK | 1980 |
| *Pseudomonas syringae* pv. *tomato* | NCPPB 878 | *S. lycopersicum* | Yugoslavia | 1961 |
|  | NCPPB 1106* | *S. lycopersicum* | UK | 1961 |
|  | NCPPB 2683 | *S. lycopersicum* | New Zealand | 1975 |
|  | NCPPB 3333 | *S. lycopersicum* | France | 1984 |
|  | NCPPB 3784 | *S. lycopersicum* | Ukraine | 1991 |
|  | NCPPB 4369 | *S. lycopersicum* | USA | 2006 |
|  | CRI 111 | *S. lycopersicum* | Czech Republic | - |
|  | CRI 211 | *S. lycopersicum* | Czech Republic | - |
| *Xanthomonas euvesicatoria* | NCPPB 941 | *Capsicum frutescens* | USA | 1961 |
|  | NCPPB 2574 | *Capsicum frutescens* | USA | 1974 |
|  | NCPPB 2594 | *Capsicum frutescens* | Brazil | 1974 |
|  | NCPPB 2968* | *Capsicum frutescens* | USA | 1977 |
| *Xanthomonas gardneri* | NCPPB 881* | *S. lycopersicum* | Yugoslavia | 1961 |
| *Xanthomonas perforans* | NCPPB 4321* | *S. lycopersicum* | USA | 2004 |
| *Xanthomonas vesicatoria* | NCPPB 422* | *S. lycopersicum* | New Zealand | 1957 |
|  | NCPPB 1421 | *S. lycopersicum* | Hungary | 1960 |
|  | NCPPB 2044 | *Physalis peruviana* | Australia | 1968 |
|  | NCPPB 3786 | *S. lycopersicum* | Ukraine | 1991 |
| strains received as *X. axonopodis* pv. *vesicatoria* |  |  |  |  |
| *Xanthomonas axonopodis* pv. *vesicatoria* | CRI 1008 | *Capsicum annuum* | Czech Republic | 2010 |
| *Xanthomonas axonopodis* pv. *vesicatoria* | CRI 1009 | *Capsicum annuum* | Czech Republic | 2010 |
| *Xanthomonas axonopodis* pv. *vesicatoria* | CRI 1011 | *Capsicum annuum* | Czech Republic | 2010 |
| *Xanthomonas axonopodis* pv. *vesicatoria* | CRI 1013 | *Capsicum annuum* | Czech Republic | 2010 |
| *Xanthomonas axonopodis* pv. *vesicatoria* | CRI 1016 | *Capsicum annuum* | Czech Republic | 2010 |
| *Xanthomonas axonopodis* pv. *vesicatoria* | CRI 1018 | *Capsicum annuum* | Czech Republic | 2010 |
| *Xanthomonas axonopodis* pv. *vesicatoria* | CRI 1023 | *Capsicum annuum* | Czech Republic | 2010 |
| *Xanthomonas axonopodis* pv. *vesicatoria* | CRI 1026 | *Capsicum annuum* | Czech Republic | 2010 |

* reference strain
