# Supplementary material for: Multiplex real-time PCR for the detection of Clavibacter michiganensis subsp. michiganensis, Pseudomonas syringae pv. tomato and pathogenic Xanthomonas species on tomato plants
Source: PLoS One. 2020 Jan 7;15(1):e0227559. doi: 10.1371/journal.pone.0227559 (PMC6946519; doi:10.1371/journal.pone.0227559)
Supplement: S2 Table — (DOC) [file pone.0227559.s003.doc]

**Supporting information**

**S2 Table. Non**target bacterial cultures used in the study.

| **Bacterium** | **Strain** | **Host** | **Origin** | **Year of isolation** |
| --- | --- | --- | --- | --- |
| *Clavibacter michiganensis* subsp.*insidiosus* | NCPPB 1109* | *Medicago sativa* | USA | 1961 |
| *Clavibacter michiganensis* subsp. *tesselarius* | NCPPB 3664* | *Triticum aestivum* | USA | 1974 |
| *Pectobacterium carotovorum* subsp. *carotovorum* | NCPPB 312* | *Solanum tuberosum* | Denmark | 1952 |
| *Pseudomonas corrugata* | NCPPB 2445* | *S. lycopersicum* | UK | 1973 |
| *Pseudomonas syringae* pv. *syringae* | NCPPB 2750 | *Syringa vulgaris* | UK | 1950 |
| *Xanthomonas axonopodis* pv*. phaseoli* | NCAIM B.01695 | *Phaseolus* sp. | Yugoslavia | - |
| *Xanthomonas campestris* pv*. armoracia* | NCAIM B.01281 | *Iberis* sp. | Tanzania | 1954 |
| *Xanthomonas campestris* pv*. campestris* | NCPPB 528* | *Brassica oleracea* var. *gemmifera* | UK | 1957 |
| *Xanthomonas campestris* pv*. incanae* | HRIW 6377 | *Matthiola incana* | USA | 1950 |
| *Xanthomonas campestris* pv*. raphani* | HRIW 8503 | *Raphanus sativus* | - | - |
| *Xanthomonas cucurbitae* | NCAIM B.01397 | *Cucurbita maxima* | New Zealand | 1968 |
| *Xanthomonas hortorum* pv*. carotae* | NCAIM B.01586 | *Daucus carota* | Yugoslavia | - |

* reference strain
