# Supplementary material for: Multiplex real-time PCR for the detection of Clavibacter michiganensis subsp. michiganensis, Pseudomonas syringae pv. tomato and pathogenic Xanthomonas species on tomato plants
Source: PLoS One. 2020 Jan 7;15(1):e0227559. doi: 10.1371/journal.pone.0227559 (PMC6946519; doi:10.1371/journal.pone.0227559)
Supplement: S3 Table — The significant similarity of nontarget organisms up to 95% is shown. (DOC) [file pone.0227559.s004.doc]

**Supporting information**

**S3 Table. In silico analysis of the specificity of designed oligonucleotides using Blastn search (NCBI/GenBank).** The significant similarity of nontarget organisms up to 95 % is shown.

| **oligonucleotide/organism** | **Max Score** | **Total Score** | **Query Cover** | **E value** | **Per. Ident** | **Accession** |
| --- | --- | --- | --- | --- | --- | --- |
| CMM-16-23S_e_fwd |  |  |  |  |  |  |
| Eukaryotic synthetic construct chromosome 13 | 38.2 | 1249 | 100% | 1.4 | 100.00% | CP034516.1 |
| Eukaryotic synthetic construct chromosome 13 | 38.2 | 1249 | 100% | 1.4 | 100.00% | CP034491.1 |
| Human DNA sequence from clone RP11-309H15 on chromosome 13, complete sequence | 38.2 | 38.2 | 95% | 1.4 | 100.00% | AL158191.17 |
| *Mus musculus* BAC clone RP23-430C3 from chromosome 18, complete sequence | 38.2 | 38.2 | 95% | 1.4 | 100.00% | AC152060.3 |
|  |  |  |  |  |  |  |
| CMM-16-23S_e_rev | Max Score | Total Score | Query Cover | E value | Per. Ident | Accession |
| *Pseudomonas syringae* strain CFBP 2116 genome assembly, chromosome: 1 | 38.2 | 38.2 | 95% | 1.4 | 100.00% | LT985192.1 |
| *Pseudomonas amygdali* pv. *morsprunorum* strain R15244 chromosome, complete genome | 38.2 | 38.2 | 95% | 1.4 | 100.00% | CP026558.1 |
| *Pseudomonas syringae* isolate CFBP3840 genome assembly, chromosome: 1 | 38.2 | 38.2 | 95% | 1.4 | 100.00% | LT963409.1 |
| *Pseudomonas syringae* pv. *cerasicola* isolate CFBP6109 genome assembly, chromosome: 1 | 38.2 | 38.2 | 95% | 1.4 | 100.00% | LT963391.1 |
| *Pseudomonas savastanoi* pv. *savastanoi* NCPPB 3335, complete genome | 38.2 | 38.2 | 95% | 1.4 | 100.00% | CP008742.1 |
|  |  |  |  |  |  |  |
| PST-hrpL_e_fwd | Max Score | Total Score | Query Cover | E value | Per. Ident | Accession |
| *Pseudomonas syringae* pv. *avii* isolate CFBP3846 genome assembly, chromosome: 1 | 40.1 | 40.1 | 100% | 0.35 | 100.00% | LT963402.1 |
| PREDICTED: *Rhagoletis zephyria* glutathione-binding protein GsiB-like (LOC108355383), partial mRNA | 38.2 | 38.2 | 95% | 1.4 | 100.00% | XM_017606545.1 |
| Zebrafish DNA sequence from clone CH211-226M7 in linkage group 11, complete sequence | 38.2 | 38.2 | 95% | 1.4 | 100.00% | BX470199.6 |
|  |  |  |  |  |  |  |
| PST-hrpL_e_rev | | | | | | |
| similarity of non-target organisms higher than 95 % were not found | |  |  |  |  |  |
|  |  |  |  |  |  |  |
| PST-hrpL_TP | Max Score | Total Score | Query Cover | E value | Per. Ident | Accession |
| *Pseudomonas syringae* strain Ps25 chromosome | 44.1 | 44.1 | 100% | 0.023 | 100.00% | CP034558.1 |
| *Pseudomonas cannabina* strain PSa1-3 *HRP* gene cluster, complete sequence | 44.1 | 44.1 | 100% | 0.023 | 100.00% | JQ517282.1 |
| *Pseudomonas syringae* pv. *maculicola* *putative sigma factor HrpL (hrpL)* gene, partial cds | 44.1 | 44.1 | 100% | 0.023 | 100.00% | U95539.1 |
| *Pseudomonas syringae* CC1557, complete sequence | 42.1 | 42.1 | 95% | 0.089 | 100.00% | CP007014.1 |
|  |  |  |  |  |  |  |
| XE_lepA_cea_rev | Max Score | Total Score | Query Cover | E value | Per. Ident | Accession |
| *Xanthomonas vasicola* pv. *vasculorum* strain Xv1601 chromosome, complete genome | 36.2 | 36.2 | 100% | 5.5 | 100.00% | CP025272.1 |
| *Xanthomonas axonopodis* pv. *phaseoli* strain ISO18C8, complete genome | 36.2 | 36.2 | 100% | 5.5 | 100.00% | CP012063.1 |
| *Xanthomonas* sp. ISO98C4, complete genome | 36.2 | 36.2 | 100% | 5.5 | 100.00% | CP012060.1 |
| *Xanthomonas axonopodis* pv. *phaseoli* strain ISO98C12, complete genome | 36.2 | 36.2 | 100% | 5.5 | 100.00% | CP012057.1 |
| *Xanthomonas axonopodis* pv. *phaseoli* strain ISO18C2, complete genome | 36.2 | 36.2 | 100% | 5.5 | 100.00% | CP012048.1 |
| *Xanthomonas* sp. strain EU1 *LepA* (*lepA*) gene, partial cds | 36.2 | 36.2 | 100% | 5.5 | 100.00% | MH068853.1 |
| *Xanthomonas campestris* pv. *arecae* strain NCPPB 2649 chromosome, complete genome* | 36.2 | 36.2 | 100% | 5.5 | 100.00% | CP034653.1 |
| *Xanthomonas vasicola* pv. *vasculorum* strain SAM119 chromosome, complete genome | 36.2 | 36.2 | 100% | 5.5 | 100.00% | CP028127.1 |
| *Xanthomonas citri* pv. *fuscans* CFBP 6988 chromosome | 36.2 | 36.2 | 100% | 5.5 | 100.00% | CP026331.1 |
| *Rhinatrema bivittatum* genome assembly, chromosome: 15 | 36.2 | 36.2 | 100% | 5.5 | 100.00% | LR584401.1 |
| *Xanthomonas axonopodis* pv. *commiphoreae* strain LMG26789 chromosome, complete genome* | 36.2 | 36.2 | 100% | 5.5 | 100.00% | CP031059.1 |
| *Xanthomonas fuscans* subsp. *fuscans* strain ISO118C5, complete genome* | 36.2 | 36.2 | 100% | 5.5 | 100.00% | CP012051.1 |
| *Xanthomonas fuscans* subsp. *fuscans* strain ISO12C3, complete genome* | 36.2 | 36.2 | 100% | 5.5 | 100.00% | CP012055.1 |
| *Xanthomonas fuscans* subsp. *fuscans* strain ISO118C1, complete genome* | 36.2 | 36.2 | 100% | 5.5 | 100.00% | CP012053.1 |
| *Xanthomonas campestris* pv. *musacearum* NCPPB 4379 chromosome, complete genome* | 36.2 | 36.2 | 100% | 5.5 | 100.00% | CP034655.1 |
| *Xanthomonas vasicola* strain NCPPB 1060 chromosome, complete genome | 36.2 | 36.2 | 100% | 5.5 | 100.00% | CP034649.1 |
| *Xanthomonas translucens* pv. *undulosa* strain ICMP11055 chromosome, complete genome | 36.2 | 36.2 | 100% | 5.5 | 100.00% | CP009750.1 |
| PREDICTED: *Capsella rubella* aspartic proteinase (LOC17895239), mRNA | 36.2 | 36.2 | 100% | 5.5 | 100.00% | XM_023787536.1 |
| *Xanthomonas citri* pv. *phaseoli* var. *fuscans* strain CFBP6167 chromosome, complete genome | 36.2 | 36.2 | 100% | 5.5 | 100.00% | CP021018.1 |
| *Xanthomonas citri* pv. *phaseoli* var. *fuscans* strain CFBP6991 chromosome, complete genome | 36.2 | 36.2 | 100% | 5.5 | 100.00% | CP021015.1 |
| *Xanthomonas citri* pv. *phaseoli* var. *fuscans* strain CFBP7767 chromosome, complete genome | 36.2 | 36.2 | 100% | 5.5 | 100.00% | CP021012.1 |
| *Xanthomonas citri* pv. *phaseoli* var. *fuscans* strain CFBP6975 chromosome, complete genome | 36.2 | 36.2 | 100% | 5.5 | 100.00% | CP021006.1 |
| *Xanthomonas citri* pv. *phaseoli* var. *fuscans* strain CFBP6166 chromosome, complete genome | 36.2 | 36.2 | 100% | 5.5 | 100.00% | CP021001.1 |
| *Xanthomonas citri* pv. *phaseoli* var. *fuscans* strain CFBP6165 chromosome, complete genome | 36.2 | 36.2 | 100% | 5.5 | 100.00% | CP020998.1 |
| *Xanthomonas citri* pv. *phaseoli* var. *fuscans* strain CFBP6996R chromosome, complete genome | 36.2 | 36.2 | 100% | 5.5 | 100.00% | CP020989.1 |
| *Xanthomonas citri* pv. *phaseoli* var. *fuscans* strain CFBP6989 chromosome, complete genome | 36.2 | 36.2 | 100% | 5.5 | 100.00% | CP020981.1 |
| *Xanthomonas axonopodis* pv. *phaseoli* strain CFBP6546R, complete genome | 36.2 | 36.2 | 100% | 5.5 | 100.00% | CP020971.1 |
|  |  |  |  |  |  |  |
| XE_lepA_aec_fwd | Max Score | Total Score | Query Cover | E value | Per. Ident | Accession |
| *Pantoea* sp. SO10 chromosome, complete genome | 36.2 | 36.2 | 100% | 5.5 | 100.00% | CP040095.1 |
| *Xanthomonas oryzae* pv. *oryzae* strain PXO61 chromosome, complete genome | 36.2 | 36.2 | 100% | 5.5 | 100.00% | CP033187.2 |
| *Cupriavidus oxalaticus* strain X32 chromosome 2, complete sequence | 36.2 | 36.2 | 100% | 5.5 | 100.00% | CP038635.1 |
| *Marinobacter hydrocarbonoclasticus* YB03 DNA, nearly complete genome | 36.2 | 36.2 | 100% | 5.5 | 100.00% | AP019537.1 |
| *Cupriavidus metallidurans* strain BS1 chromosome c1, complete sequence | 36.2 | 36.2 | 100% | 5.5 | 100.00% | CP037900.1 |
| *Xanthomonas oryzae* pv. *oryzae* strain PXO513 chromosome, complete genome | 36.2 | 36.2 | 100% | 5.5 | 100.00% | CP033188.1 |
| *Xanthomonas oryzae* pv. *oryzae* strain PXO421 chromosome, complete genome | 36.2 | 36.2 | 100% | 5.5 | 100.00% | CP033189.1 |
| *Xanthomonas oryzae* pv. *oryzae* strain PXO404 chromosome, complete genome | 36.2 | 36.2 | 100% | 5.5 | 100.00% | CP033190.1 |
| *Xanthomonas oryzae* pv. *oryzae* strain PXO364 chromosome, complete genome | 36.2 | 36.2 | 100% | 5.5 | 100.00% | CP033191.1 |
| *Xanthomonas oryzae* pv. *oryzae* strain NX0260 chromosome, complete genome | 36.2 | 36.2 | 100% | 5.5 | 100.00% | CP033192.1 |
| *Xanthomonas oryzae* pv. *oryzae* strain KXO85 chromosome, complete genome | 36.2 | 36.2 | 100% | 5.5 | 100.00% | CP033197.1 |
| *Xanthomonas oryzae* pv. *oryzae* strain JW11089 chromosome, complete genome | 36.2 | 36.2 | 100% | 5.5 | 100.00% | CP033193.1 |
| *Xanthomonas oryzae* pv. *oryzae* strain CIAT chromosome, complete genome | 36.2 | 36.2 | 100% | 5.5 | 100.00% | CP033194.1 |
| *Xanthomonas oryzae* pv. *oryzae* strain AUST2013 chromosome, complete genome | 36.2 | 36.2 | 100% | 5.5 | 100.00% | CP033196.1 |
| *Xanthomonas oryzae* strain BB151-3 chromosome, complete genome | 36.2 | 36.2 | 100% | 5.5 | 100.00% | CP036255.1 |
| *Xanthomonas oryzae* strain BAI23 chromosome, complete genome | 36.2 | 36.2 | 100% | 5.5 | 100.00% | CP036256.1 |
| *Xanthomonas oryzae* strain BB156-2 chromosome, complete genome | 36.2 | 36.2 | 100% | 5.5 | 100.00% | CP036254.1 |
| *Xanthomonas oryzae* strain NCPPB 4346 chromosome, complete genome | 36.2 | 36.2 | 100% | 5.5 | 100.00% | CP036253.1 |
| *Xanthomonas oryzae* strain NJ611 chromosome, complete genome | 36.2 | 36.2 | 100% | 5.5 | 100.00% | CP036252.1 |
| *Xanthomonas oryzae* strain X11-5A chromosome, complete genome | 36.2 | 36.2 | 100% | 5.5 | 100.00% | CP036251.1 |
| *Pseudomonas* sp. SNU WT1 chromosome, complete genome | 36.2 | 36.2 | 100% | 5.5 | 100.00% | CP035952.1 |
| *Xanthomonas vasicola* pv. *vasculorum* strain Xv1601 chromosome, complete genome | 36.2 | 36.2 | 100% | 5.5 | 100.00% | CP025272.1 |
| *Xanthomonas oryzae* pv. *oryzae* strain YN24 chromosome, complete genome | 36.2 | 36.2 | 100% | 5.5 | 100.00% | CP018089.1 |
| *Xanthomonas oryzae* pv. *oryzae* strain ScYc-b chromosome, complete genome | 36.2 | 36.2 | 100% | 5.5 | 100.00% | CP018087.1 |
| *Pseudomonas* sp. R11-23-07 chromosome, complete genome | 36.2 | 36.2 | 100% | 5.5 | 100.00% | CP027761.1 |
| *Pseudomonas* sp. R4-34-07 chromosome, complete genome | 36.2 | 36.2 | 100% | 5.5 | 100.00% | CP027760.1 |
| *Pseudomonas* sp. R2-7-07 chromosome, complete genome | 36.2 | 36.2 | 100% | 5.5 | 100.00% | CP027759.1 |
| *Pseudomonas* sp. R1-43-08 chromosome, complete genome | 36.2 | 36.2 | 100% | 5.5 | 100.00% | CP027734.1 |
| *Pseudomonas* sp. R4-39-08 chromosome, complete genome | 36.2 | 36.2 | 100% | 5.5 | 100.00% | CP027733.1 |
| *Pseudomonas* sp. R4-35-07 chromosome, complete genome | 36.2 | 36.2 | 100% | 5.5 | 100.00% | CP027732.1 |
| *Pseudomonas* sp. R2-60-08W chromosome, complete genome | 36.2 | 36.2 | 100% | 5.5 | 100.00% | CP027731.1 |
| *Pseudomonas* sp. R3-52-08 chromosome, complete genome | 36.2 | 36.2 | 100% | 5.5 | 100.00% | CP027730.1 |
| *Pseudomonas* sp. R3-18-08 chromosome, complete genome | 36.2 | 36.2 | 100% | 5.5 | 100.00% | CP027729.1 |
| *Pseudomonas* sp. R2-37-08W chromosome, complete genome | 36.2 | 36.2 | 100% | 5.5 | 100.00% | CP027728.1 |
| *Pseudomonas* sp. R5-89-07 chromosome, complete genome | 36.2 | 36.2 | 100% | 5.5 | 100.00% | CP027727.1 |
| *Pseudomonas synxantha* strain R2-54-08W chromosome, complete genome | 36.2 | 36.2 | 100% | 5.5 | 100.00% | CP027758.1 |
| *Pseudomonas synxantha* strain R2-4-08W chromosome, complete genome | 36.2 | 36.2 | 100% | 5.5 | 100.00% | CP027757.1 |
| *Pseudomonas synxantha* strain 2-79 chromosome, complete genome | 36.2 | 36.2 | 100% | 5.5 | 100.00% | CP027755.1 |
| *Xanthomonas citri* pv. *glycines* str. 8ra chromosome, complete genome | 36.2 | 36.2 | 100% | 5.5 | 100.00% | CP017188.2 |
| *Streptomyces* sp. ADI95-16 chromosome, complete genome | 36.2 | 36.2 | 100% | 5.5 | 100.00% | CP033581.1 |
| *Herbaspirillum rubrisubalbicans* strain DSM 11543 chromosome, complete genome | 36.2 | 36.2 | 100% | 5.5 | 100.00% | CP024996.1 |
| *Xanthomonas axonopodis* pv. *commiphoreae* strain LMG26789 chromosome, complete genome* | 36.2 | 36.2 | 100% | 5.5 | 100.00% | CP031059.1 |
| Sterolibacteriaceae bacterium J5B DNA, complete genome | 36.2 | 36.2 | 100% | 5.5 | 100.00% | AP018718.1 |
| *Buttiauxella* sp. 3AFRM03 chromosome, complete genome | 36.2 | 36.2 | 100% | 5.5 | 100.00% | CP033076.1 |
| *Serratia* sp. 3ACOL1 chromosome, complete genome | 36.2 | 36.2 | 100% | 5.5 | 100.00% | CP033055.1 |
| *Xanthomonas citri* pv. *citri* strain Xcc49 chromosome, complete genome | 36.2 | 36.2 | 100% | 5.5 | 100.00% | CP023662.1 |
| *Xanthomonas citri* pv. *citri* strain Xcc29-1 chromosome, complete genome | 36.2 | 36.2 | 100% | 5.5 | 100.00% | CP023661.1 |
| *Dickeya fangzhongdai* strain PA1 chromosome, complete genome | 36.2 | 36.2 | 100% | 5.5 | 100.00% | CP020872.1 |
| *Xanthomonas oryzae* pv. *oryzae* strain PXO142 chromosome, complete genome | 36.2 | 36.2 | 100% | 5.5 | 100.00% | CP031698.1 |
| *Xanthomonas oryzae* pv. *oryzae* strain ICMP3125 chromosome, complete genome | 36.2 | 36.2 | 100% | 5.5 | 100.00% | CP031697.1 |
| Sterolibacteriaceae bacterium M52 DNA, complete genome | 36.2 | 36.2 | 100% | 5.5 | 100.00% | AP019373.1 |
| *Xanthomonas fuscans* subsp. *fuscans* strain ISO118C5, complete genome* | 36.2 | 36.2 | 100% | 5.5 | 100.00% | CP012051.1 |
| *Xanthomonas axonopodis* pv. *phaseoli* strain ISO18C8, complete genome | 36.2 | 36.2 | 100% | 5.5 | 100.00% | CP012063.1 |
| *Xanthomonas* sp. ISO98C4, complete genome | 36.2 | 36.2 | 100% | 5.5 | 100.00% | CP012060.1 |
| *Xanthomonas axonopodis* pv. *phaseoli* strain ISO98C12, complete genome | 36.2 | 36.2 | 100% | 5.5 | 100.00% | CP012057.1 |
| *Xanthomonas fuscans* subsp. *fuscans* strain ISO12C3, complete genome* | 36.2 | 36.2 | 100% | 5.5 | 100.00% | CP012055.1 |
| *Xanthomonas fuscans* subsp. *fuscans* strain ISO118C1, complete genome* | 36.2 | 36.2 | 100% | 5.5 | 100.00% | CP012053.1 |
| *Xanthomonas axonopodis* pv. *phaseoli* strain ISO18C2, complete genome | 36.2 | 36.2 | 100% | 5.5 | 100.00% | CP012048.1 |
| *Xanthomonas* sp. strain EU1 *LepA* (*lepA*) gene, partial cds | 36.2 | 36.2 | 100% | 5.5 | 100.00% | MH068853.1 |
| *Xanthomonas vasicola* strain NCPPB 902 chromosome, complete genome | 36.2 | 36.2 | 100% | 5.5 | 100.00% | CP034657.1 |
| *Xanthomonas campestris* pv. *musacearum* NCPPB 4379 chromosome, complete genome* | 36.2 | 36.2 | 100% | 5.5 | 100.00% | CP034655.1 |
| *Xanthomonas campestris* pv. *arecae* strain NCPPB 2649 chromosome, complete genome* | 36.2 | 36.2 | 100% | 5.5 | 100.00% | CP034653.1 |
| *Xanthomonas vasicola* strain NCPPB 1060 chromosome, complete genome | 36.2 | 36.2 | 100% | 5.5 | 100.00% | CP034649.1 |
| *Pseudomonas mendocina* strain NCTC10897 genome assembly, chromosome: 1 | 36.2 | 36.2 | 100% | 5.5 | 100.00% | LR134290.1 |
|  |  |  |  |  |  |  |
| XE_lepA_TP | Max Score | Total Score | Query Cover | E value | Per. Ident | Accession |
| *Stenotrophomonas* sp. DAIF1 chromosome, complete genome | 36.2 | 68.4 | 100% | 5.5 | 100.00% | CP037883.1 |
| *Xanthomonas oryzae* pv. *oryzae* strain NX0260 chromosome, complete genome | 36.2 | 66.4 | 100% | 5.5 | 100.00% | CP033192.1 |
| *Xanthomonas oryzae* pv. *oryzae* strain KXO85 chromosome, complete genome | 36.2 | 66.4 | 100% | 5.5 | 100.00% | CP033197.1 |
| *Xanthomonas oryzae* pv. *oryzae* strain CIAT chromosome, complete genome | 36.2 | 66.4 | 100% | 5.5 | 100.00% | CP033194.1 |
| *Xanthomonas oryzae* pv. *oryzae* strain AUST2013 chromosome, complete genome | 36.2 | 66.4 | 100% | 5.5 | 100.00% | CP033196.1 |
| *Xanthomonas oryzae* pv. *oryzae* strain Ug11 chromosome, complete genome | 36.2 | 66.4 | 100% | 5.5 | 100.00% | CP033170.1 |
| *Xanthomonas oryzae* pv. *oryzae* strain T19 chromosome, complete genome | 36.2 | 66.4 | 100% | 5.5 | 100.00% | CP033171.1 |
| *Xanthomonas oryzae* pv. *oryzae* strain Dak16 chromosome, complete genome | 36.2 | 66.4 | 100% | 5.5 | 100.00% | CP033172.1 |
| *Xanthomonas oryzae* pv. *oryzae* strain CFBP8172 chromosome, complete genome | 36.2 | 66.4 | 100% | 5.5 | 100.00% | CP033173.1 |
| *Xanthomonas oryzae* pv. *oryzae* strain CFBP7340 chromosome, complete genome | 36.2 | 66.4 | 100% | 5.5 | 100.00% | CP033174.1 |
| *Xanthomonas oryzae* pv. *oryzae* strain CFBP7337 chromosome, complete genome | 36.2 | 66.4 | 100% | 5.5 | 100.00% | CP033175.1 |
| *Xanthomonas oryzae* pv. *oryzae* strain CFBP7325 chromosome, complete genome | 36.2 | 66.4 | 100% | 5.5 | 100.00% | CP033176.1 |
| *Xanthomonas oryzae* pv. *oryzae* strain CFBP7323 chromosome, complete genome | 36.2 | 66.4 | 100% | 5.5 | 100.00% | CP033178.1 |
| *Xanthomonas oryzae* pv. *oryzae* strain CFBP7322 chromosome, complete genome | 36.2 | 66.4 | 100% | 5.5 | 100.00% | CP033179.1 |
| *Xanthomonas oryzae* pv. *oryzae* strain CFBP7321 chromosome, complete genome | 36.2 | 66.4 | 100% | 5.5 | 100.00% | CP033180.1 |
| *Xanthomonas oryzae* pv. *oryzae* strain CFBP7320 chromosome, complete genome | 36.2 | 66.4 | 100% | 5.5 | 100.00% | CP033186.1 |
| *Xanthomonas oryzae* pv. *oryzae* strain CFBP7319 chromosome, complete genome | 36.2 | 66.4 | 100% | 5.5 | 100.00% | CP033181.1 |
| *Xanthomonas oryzae* pv. *oryzae* strain CFBP1952 chromosome, complete genome | 36.2 | 66.4 | 100% | 5.5 | 100.00% | CP033182.1 |
| *Xanthomonas oryzae* pv. *oryzae* strain CFBP1949 chromosome, complete genome | 36.2 | 66.4 | 100% | 5.5 | 100.00% | CP033184.1 |
| *Xanthomonas oryzae* pv. *oryzae* strain CIX298 chromosome, complete genome | 36.2 | 66.4 | 100% | 5.5 | 100.00% | CP036378.1 |
| *Xanthomonas oryzae* pv. *oryzae* strain CIX2374 chromosome, complete genome | 36.2 | 66.4 | 100% | 5.5 | 100.00% | CP036377.1 |
| *Xanthomonas oryzae* strain BB151-3 chromosome, complete genome | 36.2 | 66.4 | 100% | 5.5 | 100.00% | CP036255.1 |
| *Xanthomonas oryzae* strain BAI23 chromosome, complete genome | 36.2 | 66.4 | 100% | 5.5 | 100.00% | CP036256.1 |
| *Xanthomonas oryzae* strain X11-5A chromosome, complete genome | 36.2 | 66.4 | 100% | 5.5 | 100.00% | CP036251.1 |
| *Pseudomonas aeruginosa* strain B17932 chromosome, complete genome | 36.2 | 68.4 | 100% | 5.5 | 100.00% | CP034436.1 |
| *Pseudomonas aeruginosa* strain SP4527 chromosome, complete genome | 36.2 | 68.4 | 100% | 5.5 | 100.00% | CP034409.1 |
| *Pseudomonas aeruginosa* strain SP4371 chromosome, complete genome | 36.2 | 68.4 | 100% | 5.5 | 100.00% | CP034369.1 |
| *Pseudomonas aeruginosa* strain B41226 chromosome, complete genome | 36.2 | 68.4 | 100% | 5.5 | 100.00% | CP034368.1 |
| *Pseudomonas aeruginosa* strain FDAARGOS_501 chromosome, complete genome | 36.2 | 68.4 | 100% | 5.5 | 100.00% | CP033843.1 |
| *Stenotrophomonas maltophilia* strain FDAARGOS_507 chromosome, complete genome | 36.2 | 70.4 | 100% | 5.5 | 100.00% | CP033829.1 |
| *Xanthomonas axonopodis* pv. *commiphoreae* strain LMG26789 chromosome, complete genome* | 36.2 | 36.2 | 100% | 5.5 | 100.00% | CP031059.1 |
| *Pseudomonas monteilii* strain B5 chromosome, complete genome | 36.2 | 36.2 | 100% | 5.5 | 100.00% | CP022562.1 |
| *Aspergillus thermomutatus* hypothetical protein (CDV56_109141), partial mRNA | 36.2 | 36.2 | 100% | 5.5 | 100.00% | XM_026762760.1 |
| *Stenotrophomonas* sp. Pemsol chromosome | 36.2 | 68.4 | 100% | 5.5 | 100.00% | CP025780.1 |
| *Xanthomonas oryzae* pv. *oryzae* strain PXO142 chromosome, complete genome | 36.2 | 66.4 | 100% | 5.5 | 100.00% | CP031698.1 |
| *Bordetella bronchiseptica* strain I124 chromosome, complete genome | 36.2 | 36.2 | 100% | 5.5 | 100.00% | CP025069.1 |
| *Xanthomonas fuscans* subsp. *fuscans* strain ISO118C5, complete genome* | 36.2 | 36.2 | 100% | 5.5 | 100.00% | CP012051.1 |
| *Xanthomonas fuscans* subsp. *fuscans* strain ISO12C3, complete genome* | 36.2 | 36.2 | 100% | 5.5 | 100.00% | CP012055.1 |
| *Xanthomonas fuscans* subsp. *fuscans* strain ISO118C1, complete genome* | 36.2 | 36.2 | 100% | 5.5 | 100.00% | CP012053.1 |
| *Xanthomonas campestris* pv. *musacearum* NCPPB 4379 chromosome, complete genome* | 36.2 | 36.2 | 100% | 5.5 | 100.00% | CP034655.1 |
| *Xanthomonas vasicola* strain NCPPB 1060 chromosome, complete genome | 36.2 | 36.2 | 100% | 5.5 | 100.00% | CP034649.1 |
| *Xanthomonas oryzae* pv. *oryzae* strain PXO61 chromosome, complete genome | 36.2 | 66.4 | 100% | 5.5 | 100.00% | CP033187.2 |
| *Pseudomonas aeruginosa* strain PABL048 chromosome, complete genome | 36.2 | 68.4 | 100% | 5.5 | 100.00% | CP039293.1 |
| *Xanthomonas oryzae* pv. *oryzae* strain PXO513 chromosome, complete genome | 36.2 | 66.4 | 100% | 5.5 | 100.00% | CP033188.1 |
| *Xanthomonas oryzae* pv. *oryzae* strain PXO421 chromosome, complete genome | 36.2 | 66.4 | 100% | 5.5 | 100.00% | CP033189.1 |
| *Xanthomonas oryzae* pv. *oryzae* strain PXO404 chromosome, complete genome | 36.2 | 66.4 | 100% | 5.5 | 100.00% | CP033190.1 |
| *Xanthomonas oryzae* pv. *oryzae* strain PXO364 chromosome, complete genome | 36.2 | 66.4 | 100% | 5.5 | 100.00% | CP033191.1 |
| *Xanthomonas oryzae* pv. *oryzae* strain JW11089 chromosome, complete genome | 36.2 | 66.4 | 100% | 5.5 | 100.00% | CP033193.1 |
| *Xanthomonas oryzae* pv. *oryzae* strain CFBP7324 chromosome, complete genome | 36.2 | 66.4 | 100% | 5.5 | 100.00% | CP033177.1 |
| *Xanthomonas oryzae* pv. *oryzae* strain CFBP1951 chromosome, complete genome | 36.2 | 66.4 | 100% | 5.5 | 100.00% | CP033183.1 |
| *Xanthomonas oryzae* pv. *oryzae* strain CFBP1948 chromosome, complete genome | 36.2 | 66.4 | 100% | 5.5 | 100.00% | CP033185.1 |
| *Stenotrophomonas maltophilia* strain SVIA2 chromosome | 36.2 | 70.4 | 100% | 5.5 | 100.00% | CP033586.1 |
| *Pseudomonas aeruginosa* strain PA-VAP-3 chromosome | 36.2 | 68.4 | 100% | 5.5 | 100.00% | CP028330.1 |
| *Pseudomonas aeruginosa* strain PA-VAP-4 chromosome | 36.2 | 98.6 | 100% | 5.5 | 100.00% | CP028368.1 |
| *Xanthomonas oryzae* strain BB156-2 chromosome, complete genome | 36.2 | 66.4 | 100% | 5.5 | 100.00% | CP036254.1 |
| *Xanthomonas oryzae* strain NCPPB 4346 chromosome, complete genome | 36.2 | 66.4 | 100% | 5.5 | 100.00% | CP036253.1 |
| *Xanthomonas oryzae* strain NJ611 chromosome, complete genome | 36.2 | 66.4 | 100% | 5.5 | 100.00% | CP036252.1 |
| *Xanthomonas hortorum* pv. *hederae* strain Hed1 *LepA* (*lepA*) gene, partial cds | 36.2 | 36.2 | 100% | 5.5 | 100.00% | MK124762.1 |
| *Pseudomonas aeruginosa* strain SP2230 chromosome, complete genome | 36.2 | 68.4 | 100% | 5.5 | 100.00% | CP034434.1 |
| *Pseudomonas aeruginosa* strain B14130 chromosome, complete genome | 36.2 | 68.4 | 100% | 5.5 | 100.00% | CP034435.1 |
| *Xanthomonas vasicola* pv. *vasculorum* strain Xv1601 chromosome, complete genome | 36.2 | 36.2 | 100% | 5.5 | 100.00% | CP025272.1 |
| *Pseudomonas entomophila* strain 2014 chromosome, complete genome | 36.2 | 36.2 | 100% | 5.5 | 100.00% | CP034337.1 |
| *Xanthomonas oryzae* pv. *oryzae* strain YN24 chromosome, complete genome | 36.2 | 66.4 | 100% | 5.5 | 100.00% | CP018089.1 |
| *Xanthomonas oryzae* pv. *oryzae* strain ScYc-b chromosome, complete genome | 36.2 | 66.4 | 100% | 5.5 | 100.00% | CP018087.1 |
| *Xanthomonas citri* pv. *glycines* str. 8ra chromosome, complete genome | 36.2 | 36.2 | 100% | 5.5 | 100.00% | CP017188.2 |
| *Pseudomonas aeruginosa* strain FDAARGOS_571 chromosome, complete genome | 36.2 | 68.4 | 100% | 5.5 | 100.00% | CP033833.1 |
| *Pseudomonas aeruginosa* strain H25883 chromosome, complete genome | 36.2 | 68.4 | 100% | 5.5 | 100.00% | CP033686.1 |
| *Pseudomonas aeruginosa* strain H26023 chromosome, complete genome | 36.2 | 68.4 | 100% | 5.5 | 100.00% | CP033685.1 |
| *Pseudomonas aeruginosa* strain SP4528 chromosome, complete genome | 36.2 | 68.4 | 100% | 5.5 | 100.00% | CP033439.1 |
| *Pseudomonas aeruginosa* strain BA15561 chromosome, complete genome | 36.2 | 68.4 | 100% | 5.5 | 100.00% | CP033432.1 |
| *Pseudomonas* sp. LTGT-11-2Z chromosome, complete genome | 36.2 | 36.2 | 100% | 5.5 | 100.00% | CP033104.1 |
| *Xanthomonas citri* pv. *citri* strain Xcc49 chromosome, complete genome | 36.2 | 36.2 | 100% | 5.5 | 100.00% | CP023662.1 |
| *Xanthomonas citri* pv. *citri* strain Xcc29-1 chromosome, complete genome | 36.2 | 36.2 | 100% | 5.5 | 100.00% | CP023661.1 |
| *Pseudomonas aeruginosa* strain 268 chromosome, complete genome | 36.2 | 68.4 | 100% | 5.5 | 100.00% | CP032761.1 |
| *Xanthomonas perforans* strain GEV2489 GTP-binding protein (*lepA*) gene, partial cds | 36.2 | 36.2 | 100% | 5.5 | 100.00% | MH550123.1 |
| *Pseudomonas aeruginosa* strain BA7823 chromosome, complete genome | 36.2 | 68.4 | 100% | 5.5 | 100.00% | CP032569.1 |
| *Xanthomonas oryzae* pv. *oryzae* strain ICMP3125 chromosome, complete genome | 36.2 | 66.4 | 100% | 5.5 | 100.00% | CP031697.1 |
| *Pseudomonas aeruginosa* strain GIMC5016:PA1840, complete sequence | 36.2 | 68.4 | 100% | 5.5 | 100.00% | CP034430.1 |
| *Xanthomonas* sp. strain EU1 *LepA* (*lepA*) gene, partial cds | 36.2 | 36.2 | 100% | 5.5 | 100.00% | MH068853.1 |
| *Xanthomonas vasicola* strain NCPPB 902 chromosome, complete genome | 36.2 | 36.2 | 100% | 5.5 | 100.00% | CP034657.1 |
| *Xanthomonas campestris* pv. *arecae* strain NCPPB 2649 chromosome, complete genome* | 36.2 | 36.2 | 100% | 5.5 | 100.00% | CP034653.1 |
| *Pseudomonas aeruginosa* strain NCTC13715 genome assembly, chromosome: 1 | 36.2 | 68.4 | 100% | 5.5 | 100.00% | LR134330.1 |
| *Stenotrophomonas maltophilia* strain NCTC13014 genome assembly, chromosome: 1 | 36.2 | 100.0 | 100% | 5.5 | 100.00% | LR134301.1 |
| *Pseudomonas mendocina* strain NCTC10897 genome assembly, chromosome: 1 | 36.2 | 36.2 | 100% | 5.5 | 100.00% | LR134290.1 |

* Potential source of a false positive result.
