# Supplementary material for: Multiplex real-time PCR for the detection of Clavibacter michiganensis subsp. michiganensis, Pseudomonas syringae pv. tomato and pathogenic Xanthomonas species on tomato plants
Source: PLoS One. 2020 Jan 7;15(1):e0227559. doi: 10.1371/journal.pone.0227559 (PMC6946519; doi:10.1371/journal.pone.0227559)
Supplement: S4 Table — (DOC) [file pone.0227559.s005.doc]

**Supporting information**

**S4 Table. Ct values (mean value ± SD) obtained for simplex real-time PCRs.**

| bacterium | strain | HEX | FAM | Cy5 |
| --- | --- | --- | --- | --- |
| *target bacteria* | | | | |
| Cmm | NCPPB 515 | 15.69 ± 0.02 | - | - |
|  | NCPPB 1064 | 16.68 ± 0.09 | - | - |
|  | NCPPB 1496 | 15.80 ± 0.07 | - | - |
|  | NCPPB 2323 | 16.78 ± 0.04 | - | - |
|  | NCPPB 2979* | 16.97 ± 0.03 | - | - |
|  | NCPPB 3120 | 16.64 ± 0.12 | - | - |
| Pst | NCPPB 878 | - | - | - |
|  | NCPPB 1106* | - | 16.82 ± 0.03 | - |
|  | NCPPB 2683 | - | 17.00 ± 0.20 | - |
|  | NCPPB 3333 | - | 18.40 ± 0.07 | - |
|  | NCPPB 3784 | - | 17.95 ± 0.15 | - |
|  | NCPPB 4369 | - | 17.77 ± 0.04 | - |
|  | CRI 111 | - | 18.09 ± 0.16 | - |
|  | CRI 211 | - | 18.42 ± 0.06 | - |
| Xe | NCPPB 941 | - | - | 17.91 ± 0.05 |
|  | NCPPB 2574 | - | - | 24.66 ± 0.30 |
|  | NCPPB 2594 | - | - | 24.46 ± 0.10 |
|  | NCPPB 2968* | - | - | 21.32 ± 0.04 |
| Xv | NCPPB 422* | - | - | 23.69 ± 0.04 |
|  | NCPPB 1421 | - | - | 20.81 ± 0.16 |
|  | NCPPB 2044 | - | - | 19.43 ± 0.41 |
|  | NCPPB 3786 | - | - | 22.41 ± 0.12 |
| Xg | NCPPB 881* | - | - | 22.27 ± 0.14 |
| Xp | NCPPB 4321* | - | - | 18.87 ± 0.06 |
| Xap | CRI 1008 | - | - | 24.75 ± 0.21 |
|  | CRI 1009 | - | - | 24.63 ± 0.11 |
|  | CRI 1011 | - | - | 22.46 ± 0.28 |
|  | CRI 1013 | - | - | 20.17 ± 0.63 |
|  | CRI 1016 | - | - | 20.96 ± 0.85 |
|  | CRI 1018 | - | - | 19.58 ± 0.06 |
|  | CRI 1023 | - | - | 23.63 ± 0.30 |
|  | CRI 1026 | - | - | 22.19 ± 0.44 |
| *other bacteria* | | | | |
| Cmi | NCPPB 1109* | - | - | - |
| Cmt | NCPPB 3664* | - | - | - |
| Pcc | NCPPB 312* | - | - | - |
| Pc | NCPPB 2445* | - | - | - |
| Pss | NCPPB 2750 | - | - | - |
| Xap | NCAIM B.01695 | - | - | 22.15 ± 0.09 |
| Xca | NCAIM B.01281 | - | - | - |
| Xcc | NCPPB 528* | - | - | - |
| Xci | HRIW 6377 | - | - | - |
| Xcr | HRIW 8503 | - | - | - |
| Xcu | NCAIM B.01397 | - | - | - |
| Xhc | NCAIM B.01586 | - | - | 23.43 ± 0.04 |

* reference strain
